# Silencing of Apoptosis-Inducing factor and poly(ADP-ribose) glycohydrolase reveals novel roles in breast cancer cell death after chemotherapy

**DOI:** 10.1186/1476-4598-11-48

**Published:** 2012-07-29

**Authors:** Xiaoxing Feng, Yiran Zhou, Alicia M Proctor, Mandi M Hopkins, Mengwei Liu, David W Koh

**Affiliations:** 1 Department of Pharmaceutical Sciences, College of Pharmacy, Washington State University, P.O. Box 646534, Pullman, WA, 99164-6534, USA

**Keywords:** Poly(ADP-ribose), Apoptosis-inducing factor, Caspase-independent cell death, PARG, Breast cancer, RNA interference

## Abstract

**Background:**

Cell death induced by poly(ADP-ribose) (PAR) and mediated by apoptosis-inducing factor (AIF) is well-characterized in models of ischemic tissue injury, but their roles in cancer cell death after chemotherapy are less understood.

**Methods:**

Here we investigated the roles of PAR and AIF by RNA interference (RNAi) in MDA-MB-231 and MCF-7 breast adenocarcinoma cells after chemotherapy. Differences in effects were statistically tested by analysis-of-variance and unpaired student’s t-test.

**Results:**

Silencing of AIF by RNAi led to decreased MDA-MB-231 and MCF-7 breast cancer cell death after chemotherapy, which demonstrates a critical role for AIF. RNAi silencing of PAR glycohydrolase (PARG), the primary enzyme that catalyzes the hydrolysis of PAR, led to increased PAR levels but decreased cell death. Further investigation into the possible role of PAR in apoptosis revealed decreased caspase-3/7/8/9 activity in PARG-null cells. Interestingly, the pharmacologic inhibition of caspase activity in PARG-silenced breast cancer cells led to increased cell death after chemotherapy, which indicates that an alternative cell death pathway is activated due to elevated PAR levels and caspase inhibition. AIF silencing in these cells led to profound protection from chemotherapy, which demonstrates that the increased cell death after PARG silencing and caspase inhibition was mediated by AIF.

**Conclusions:**

The results show a role for AIF in breast cancer cell death after chemotherapy, the ability of PAR to regulate caspase activity, and the ability of AIF to substitute as a primary mediator of breast cancer cell death in the absence of caspases. Thus, the induction of cell death by PAR/AIF may represent a novel strategy to optimize the eradication of breast tumors by activating an alternative cell death pathway.

## Background

The ability to induce alternative pathways of cell death in tumors after chemotherapy is potentially an innovative strategy to optimize cancer cell death. Such optimization could lead to the improved eradication of tumors or the successful chemotherapeutic treatment of cancer cells that evade apoptotic cell death. One controversial, yet potentially novel, cell death pathway is poly(ADP-ribose) (PAR)-dependent cell death mediated by apoptosis-inducing factor (AIF). The synthesis of PAR by the PAR polymerases (PARPs) [1] is essential for the maintenance of genomic integrity after cell stress. In the absence of stress, AIF has a physiologic role in the mitochondria, as it contains NADH oxidase activity [2] and it is involved in oxidative phosphorylation [3]. However, therapeutic doses of DNA-damaging chemotherapeutic agents activate the synthesis of PAR and ultimately leads to the translocation of AIF from the mitochondria to the nucleus [4]. Once AIF translocates to the nucleus, it has a cytotoxic role, where it becomes part of a DNA fragmenting complex that initiates caspase-independent cell death [5]. Thus, AIF is also a pro-cell death mediator for a unique programmed cell death pathway known as “necroptosis” [6].

Although some of the downstream cellular morphological changes in AIF-mediated cell death are similar to those characteristic of apoptosis, such as chromatin condensation, DNA fragmentation, and phosphatidylserine exposure [7], a unique feature of this cell death pathway is that it can occur in the absence of caspases [8]. In fact, AIF was the first protein reported to mediate a caspase-independent form of programmed cell death [7]. This observation remains controversial, as initial studies demonstrated AIF-mediated cell death to be caspase-independent [4,7], while later studies demonstrated it to be dependent of caspases [9,10]. To date, cell death studies support the possibility that AIF contributes to caspase-dependent cell death and it induces an alternative form of apoptosis that is caspase-independent [11].

Another unique feature of AIF-mediated cell death is its dependence on the synthesis of PAR. Studies have shown that AIF fails to translocate to the nucleus in the absence of PAR synthesis [12,13]. We recently showed that the genetic disruption of PAR glycohydrolase (PARG), the primary enzyme that catalyzes hydrolysis of PAR [14], leads to increased levels of PAR and cell death mediated by AIF [15]. Further, PAR was shown to bind AIF, which leads to its release from the mitochondria [16]. PAR is primarily synthesized in the nucleus by the PARPs in response to DNA damage, and PAR causes the mitochondrial release of AIF. These observations suggest that nuclear PAR translocates to the mitochondria and directly acts as the signal for AIF release. This PAR-dependent cell death that is mediated by AIF is termed “parthanatos” [17].

Current studies, however, suggest that PAR-induced cell death mediated by AIF is only important in certain cell types. For example, studies support a critical role for AIF in the death of post-mitotic cells due to pathologic conditions, such as tissue injury that occurs secondary to ischemia [18], reperfusion injury [13], and various conditions exacerbated by inflammation [19,20].The role of AIF in cancer cell death after chemotherapy is less understood. Studies indicate that chemotherapeutic agents induce both caspase-dependent and caspase-independent cell death in cancer cells [21,22], and that AIF plays a critical role in the cell death of only a few types of tumor cells [23-25]. Thus, it is unknown if AIF can serve as a primary mediator of cell death in breast cancer cells after chemotherapeutic treatments.

Here, we show a significant role for AIF-mediated cell death induced by PAR in the death of invasive breast cancer cells. Further, we demonstrate that AIF can substitute as caspase executioner in these cells in response to chemotherapy and in the absence of caspase activity. In addition, we show that the inability to catalyze the hydrolysis of PAR negatively regulates caspase activity, which provides further insight into the ability of PAR and AIF to cause cell death in cancer cells in the absence of caspases. Thus, cell death initiated by PAR and mediated by AIF is potentially a novel strategy to induce an alternative pathway of cell death in breast tumors.

## Results

### Silencing of AIF by RNAi leads to decreased nuclear translocation of AIF after DNA damage

**Figure 1:**
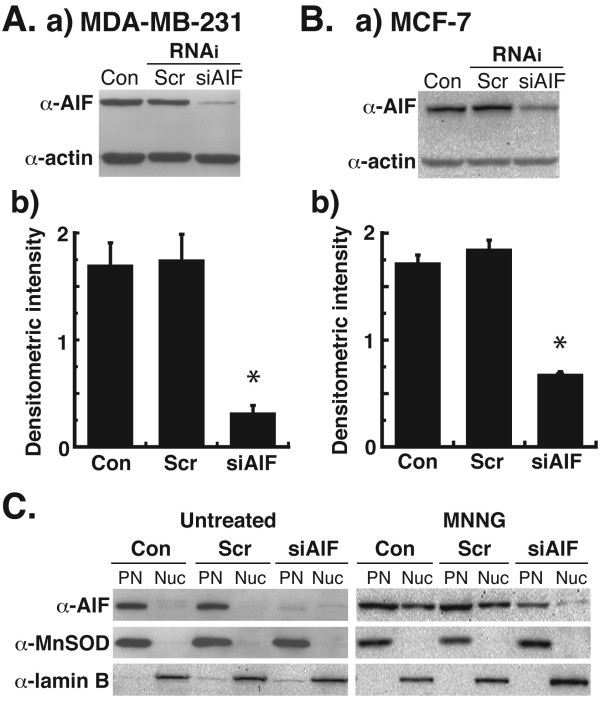
**Silencing of apoptosis-inducing factor (AIF) by siRNA in human breast adenocarcinoma cells.** Human breast adenocarcinoma cells were transfected with 20 nM small interfering RNA (siRNA) oligos for AIF as previously reported [26]. **A**, (a) Immunoblotting detection of AIF in MDA-MB-231 cell extracts 48 h after siRNA transfection using polyclonal anti-AIF antibody (Rockland Immunochemcials) (1:1000 dilution). Controls were provided by untransfected cells (Con) and cells transfected with scrambled siRNA oligos (Scr). Densitometric quantification (b) of AIF protein bands from (a) was then performed. Values represent AIF/actin ratios of the densitometric intensities of the protein bands. *P < 0.01 between Scr and AIF (one-way ANOVA and unpaired Student’s t test). Error bars represent the standard error of the mean (SEM). **B**, Immunoblotting detection (a) and densitometric quantification of AIF in MCF-7 cell extracts 48 h after siRNA transfection. **C**, MDA-MB-231 cells were transfected with siRNA oligos, treated with 0.5 mM N-methyl-N’-nitro-N-nitrosoguanidine (MNNG) for 45 min, then analyzed by immunoblot for levels of nuclear AIF. Controls for cell fractionations were provided by mitochondrial manganese superoxide dismutase (MnSOD) and nuclear lamin B2. PN, post-nuclear fraction (cytoplasm + mitochondria); Nuc, nuclear fraction. All experiments were repeated at least three times with similar results.

Because the genetic disruption of the AIF gene causes embryonic lethality [4], no AIF-null breast cancer cells are available. However, using the previously reported protocol to silence the expression of AIF in melanocytes by RNAi [26], we successfully decreased AIF levels in human breast adenocarcinoma cells (Figure 1). Following AIF RNAi oligo treatment, AIF protein levels were significantly decreased after 48 h in MDA-MB-231 (Figure 1A-a) and MCF-7 cells (Figure 1B-a). Densitometric analysis of protein levels indicated greater than 80% knockdown of AIF in MDA-MB-231 cells (Figure 1A-b) and approximately 65% knockdown of AIF in MCF-7 cells (Figure 1B-b) after RNAi treatment as compared to untransfected (Con) or scrambled (Scr) RNAi controls. Because AIF translocates from the mitochondria to the nucleus following cytotoxic doses of the DNA-methylating agent, N-methyl-N’-nitro-N-nitrosoguanidine (MNNG) [12], we next analyzed levels of AIF in the nuclei of MDA-MB-231 cells after MNNG treatment. No AIF was observed in the nuclei of untreated cells, but a dose of 0.5 mM MNNG x45 min led to significant levels of AIF in the nuclei of control and scrambled RNAi-treated cells (Figure 1C). However, nuclear AIF in AIF-silenced cells was significantly decreased. These results demonstrate that RNAi silencing of AIF in breast adenocarcinoma cells leads to decreased AIF levels and decreased levels of nuclear AIF after treatment with a cytotoxic DNA-damaging agent. 

### Silencing of AIF by RNAi leads to decreased cell death after treatment with DNA-damaging chemotherapeutic agents

**Figure 2:**
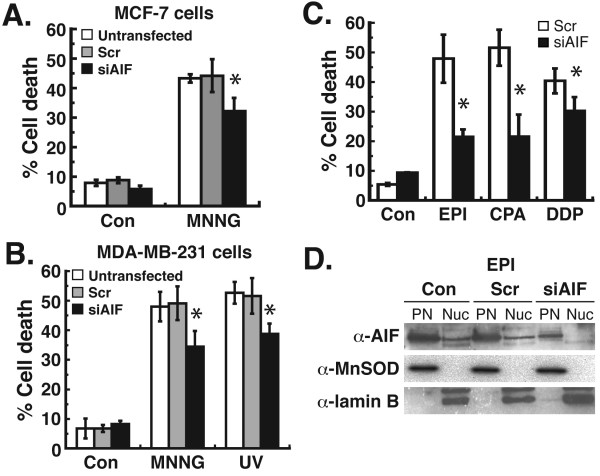
**Chemotherapeutic treatment of human breast adenocarcinoma cells after the silencing of AIF.** Cells were transfected with siRNA for 48 h as before. **A**, MCF-7 cells were then treated with 0.5 mM MNNG x45 min. **B**, MDA-MB-231 cells were then treated with MNNG or 25 J/m^2^ UV-C radiation. After 24 h, cell death was quantified by FACS after propidium iodide (PI)/annexin V-FITC staining. **C**, MDA-MB-231 cells were treated with 20 μM epirubicin (EPI) x6 h, 10 mM cyclophosphamide (CPA) x24 h, or 0.5 mM cisplatin (DDP) x6 h after siRNA transfection. Cell death was then quantified by FACS after PI/annexin staining. For **A-C**, *P < 0.01 between Scr and AIF (one-way ANOVA and unpaired Student’s t test). Error bars represent the SEM. **D**, Analysis of nuclear AIF levels in siRNA transfected cells treated with EPI. Con, untransfected cells; Scr, scrambled siRNA transfected cells. All experiments were repeated at least twice with similar results.

We next analyzed the effect of AIF silencing on cell death in MDA-MB-231 and MCF-7 cells after treatment with chemotherapeutic agents. MNNG and UV radiation are two DNA-damaging treatments known to induce PAR synthesis and AIF-mediated cell death [12,15,27]. The silencing of AIF by RNAi led to a 27% decrease in cell death after MNNG treatment in MCF-7 cells (Figure 2A). In MDA-MB-231 cells after AIF RNAi, a 30% decrease in cell death was observed after MNNG treatment and a 25% reduction was observed after treatment with UV radiation (Figure 2B). These results indicate a significant role for AIF in breast adenocarcinoma cells after DNA-damaging treatments. We then treated MDA-MB-231 cells with the chemotherapeutic agents epirubicin (EPI), cyclophosphamide (CPA), or cisplatin (DDP), all of which are utilized in treating breast cancer [28,29]. AIF silencing led to a 25% reduction in cell death after DDP treatment (Figure 2C). However, a more profound reduction in cell death was observed after EPI or CPA treatment, where AIF knockdown led to a 55-60% reduction in cell death. To confirm that these effects were mediated by a decrease in the pro-cell death function of AIF, nuclear AIF levels were analyzed in these cells. The results demonstrate no significant levels of AIF in the nuclei of MDA-MB-231 cells after AIF silencing and EPI treatment (Figure 2D), which indicates that the protective effects observed after AIF silencing were due to decreased levels of AIF-mediated cell death. Taken together, these results demonstrate an important role for AIF in the death of breast adenocarcinoma cells after chemotherapy. 

### Silencing of PARG by RNAi leads to increased and prolonged levels of PAR after treatment with DNA-damaging agents

**Figure 3:**
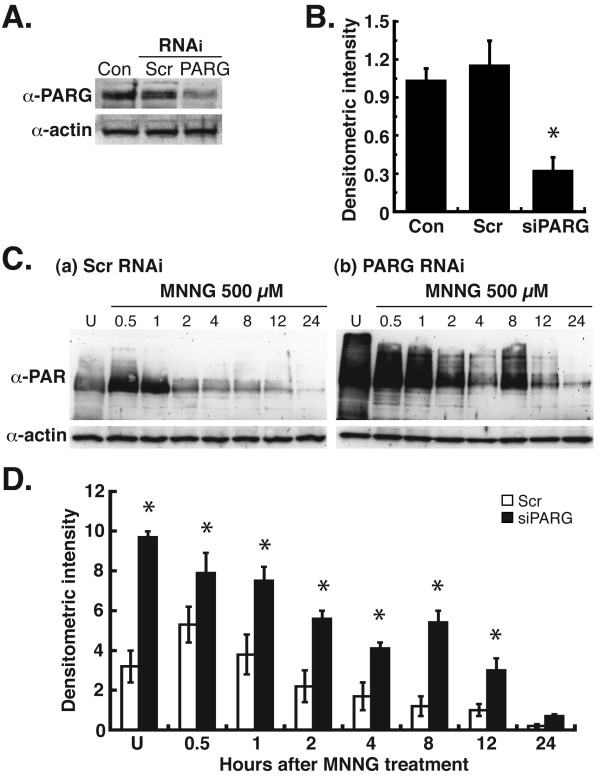
**Silencing of poly(ADP-ribose) glycohydrolase (PARG) by siRNA in MDA-MB-231 cells.** MDA-MB-231 cells were transfected with siRNA oligos for PARG as previously reported [38]. **A**, Immunoblotting detection of PARG in cell extracts 48 h after transfection using polyclonal anti-PARG antibody (Millipore) (1:2,000 dilution). Equivalent controls wereutilized as in Fig. 1A. **B**, Densitometric quantification of PARG protein bands from **A**. *P < 0.01 between Scr and AIF (one-way ANOVA and unpaired Student’s t test). Error bars represent the SEM. **C**, MDA-MB-231 cells were transfected with PARG siRNA oligos, treated with 0.5 mM MNNG, then analyzed for levels of PAR by immunoblot from 0.5-24 h. Equal protein loading per lane was verified by immunoblotting detection of β-actin. **D**, Densitometric quantification of PAR levels from **C**. *P < 0.01 between Scr and PARG (one-way ANOVA and unpaired Student’s t-test). Error bars represent the SEM.

The ability of AIF to mediate cell death is dependent on the synthesis of PAR. Also, our previous studies demonstrated that treatment of PARG-null cells with DNA-damaging agents led to increased levels of PAR and cell death mediated by AIF [15]. Thus, we next investigated the effects of the silencing of PARG, the primary enzyme that catalyzes the hydrolysis of PAR, on PAR levels in MDA-MB-231 breast cancer cells. The results demonstrated that the silencing of PARG by RNAi decreased PARG levels approximately 70% in MDA-MB-231 cells (Figure 3A and B). Analysis of PAR levels after MNNG treatment in control cells demonstrated peak levels of PAR at 0.5 h, with a decrease in PAR levels from 1–8 h thereafter (Figure 3C, a). In PARG silenced cells, however, a high level of PAR was observed in untreated cells (Figure 3C, b). After MNNG treatment, PAR levels decreased from 0.5 – 4 h, which indicates PARG activity. However, at all time points except 24 h, PAR levels were significantly elevated in PARG silenced MDA-MB-231 cells as compared to control cells (Figure 3D). These results thus demonstrate that RNAi silencing of PARG leads to increased levels of PAR in MDA-MB-231 breast cancer cells after treatment with cytotoxic doses of the DNA-damaging agent, MNNG. 

### Silencing of PARG by RNAi leads to decreased levels of cell death and increased levels of procaspase-8 after DNA damage

**Figure 4:**
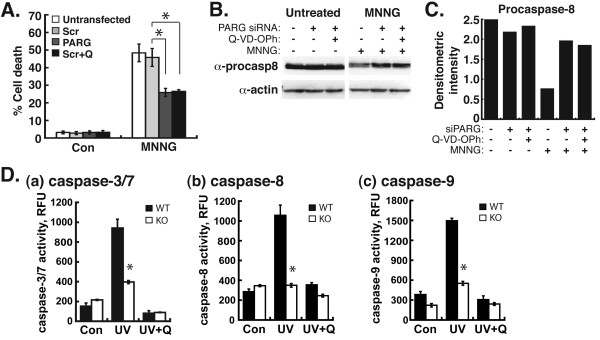
**Analysis of cell death and caspases in MDA-MB-231 cells after PARG silencing or genetic disruption. A**, MDA-MB-231 cells pretreated with the pan-caspase inhibitor, Q-VD-OPH (Q), were transfected with PARG siRNA. 48 h after transfection, cells were treated with 0.5 mM MNNG for 45 min, and 24 h later cell death was quantified by FACS. All error bars represent the SEM. *P < 0.01 between Scr and PARG or Scr and Scr + Q (one-way ANOVA and unpaired Student’s t-test). **B**, extracts from cells in **A** were probed for the immunoblotting detection of procaspase-8 levels. **C**, Procaspase-8 protein levels in **B** were quantified by densitometric analysis. Values represent procaspase-8/actin ratios of the densitometric intensities of the protein bands. **D**, Wild-type (WT) and PARG-null (KO) cells were treated with 25 J/m^2^ UV-C radiation and 12 h later caspase-3 (a), caspase-8 (b), and caspase-9 (c) activity was assayed. Q, pretreatment with Q-VD-OPh 30 min prior to UV treatment and until caspase activity assays. Error bars represent the SEM. *P < 0.01 between WT and KO (one-way ANOVA and unpaired Student’s t-test). RFU, relative fluorescence unit.

We next determined the effect of PARG silencing on cell death in MDA-MB-231 cells. Surprisingly, PARG silencing led to a 43% decrease in cell death after MNNG treatment (Figure 4A). In the same experiment, control cells pretreated with Q-VD-OPh, a pan-caspase inhibitor [30], also led to a 43% decrease in cell death in MDA-MB-231 cells after MNNG treatment (Figure 4A). Because of the similarities, we next analyzed the effect of PARG knockdown or the genetic disruption of PARG on caspase activity and and procaspase levels in these cells. Procaspase-8 is an initiator caspase that must be activated by proteolytic cleavage [31]. Following PARG silencing by RNAi in MDA-MB-231 cells, increased levels of procaspase-8 were observed as compared to untransfected control cells after MNNG treatment (Figure 4B). Quantification of procaspase-8 protein levels by densitometric analysis confirmed this observation (Figure 4C). This level of procaspase-8 in PARG-silenced cells was similar to the level observed after pretreatment with Q-VD-OPh, which suggests that the silencing of PARG led to decreased activation of procaspase-8 after MNNG treatment. This possible effect of PARG silencing on procaspase-8 levels prompted us to investigate the effect of the genetic deletion of PARG on caspase activity. We previously demonstrated increased levels of PAR and decreased levels of caspase-3/7 activity in PARG-null cells after UV treatment [15]. We next investigated caspase-8 and −9 activity in PARG-null cells after treatment with UV radiation. Previously, UV-C radiation was shown to activate both intrinsic and extrinsic pathways of apoptosis [32]. Thus, this treatment is sufficient to characterize caspase-8 and −9 activity, the initiator caspases for the extrinsic and intrinsic apoptotic pathways, respectively. As the positive control for decreased caspase activity, we again observed decreased caspase-3/7 activity in PARG-null cells after UV-C radiation (Fig. 4D-a). However, we also observed decreased levels of caspase-8 and caspase-9 activity in PARG-null cells after UV-C treatment (Figure 4D-b and c). These decreased levels of caspase-8/9 activity were similar to the levels observed after PARG-null cells were pretreated with Q-VD-OPh, which indicates that increased levels of PAR due to the absence of PARG leads to the negative regulation of caspase-8/9 activity. These results thus demonstrate that the absence of PARG leads to decreased caspase-3, -7, -8, and −9 activity after DNA-damaging treatments. Taken together, these results suggest that the knockdown or genetic deletion of PARG, which both lead to increased levels of PAR, leads to decreased caspase activity after DNA damage. Further, they suggest that the decrease in cell death observed in PARG-silenced cells after MNNG treatment was due to the inhibition of caspase-mediated apoptotic cell death by elevated levels of PAR.Thus, the hydrolysis of PAR by PARG appears to have a role in regulating caspase activity after DNA-damaging treatments in breast adenocarcinoma cells. 

### The silencing of PARG in combination with the inhibition of caspases leads to increased cell death in MDA-MB-231 cells after MNNG treatment that is mediated by AIF

**Figure 5:**
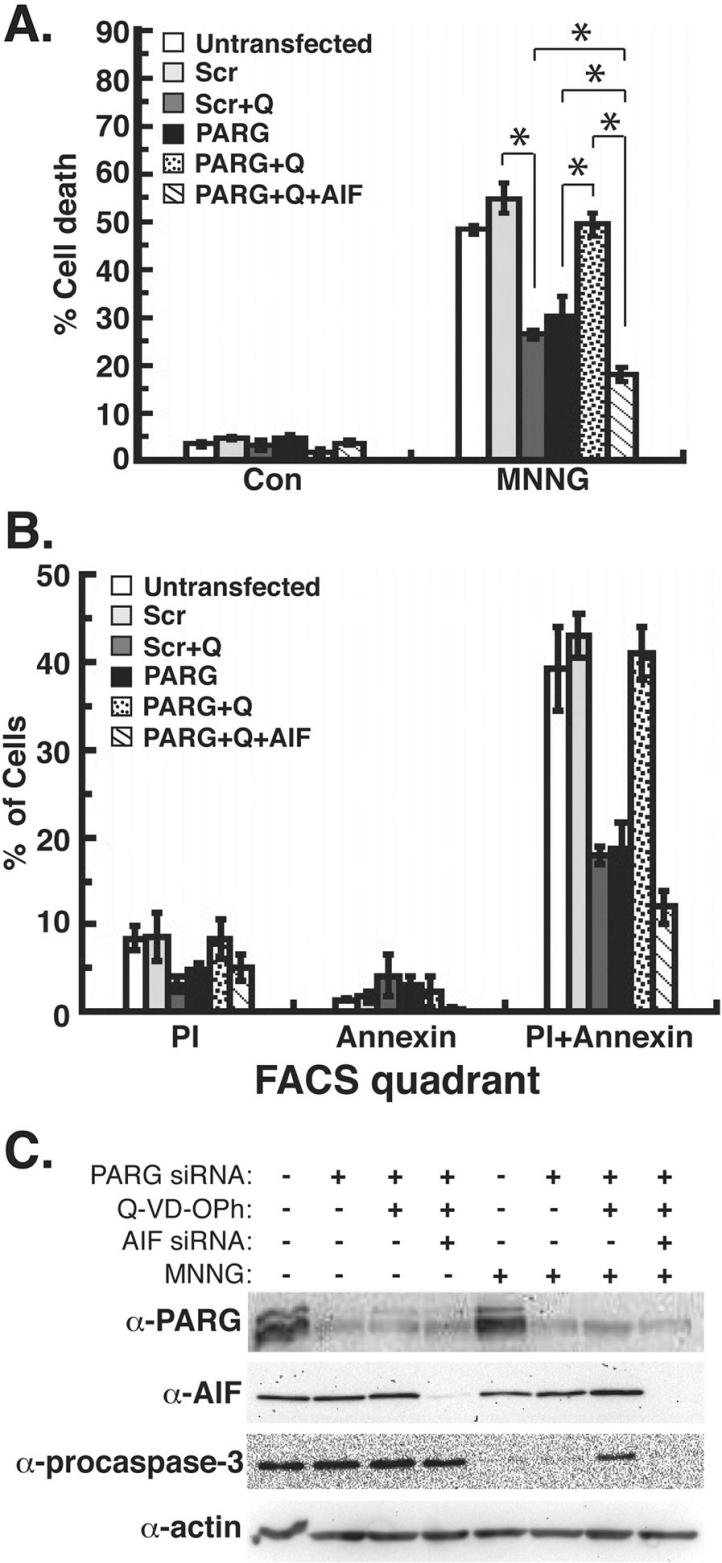
**MDA-MB-231 cell death after silencing of PARG, inhibition of caspases, and RNAi knockdown of AIF.** MDA-MB-231 cells were pretreated with Q-VD-OPh and transfected with siRNA for PARG and AIF. **A**, Cell death was quantified by FACS after treatment with 0.5 mM MNNG for 45 min. **B**, The percentage of cells that exhibited propidium iodide (PI), annexin V-FITC, or dual PI/annexin V-FITC fluorescence from FACS analysis in **B** were determined. **C**, Extracts from cells in **A** were probed for the immunoblotting detection of PARG, AIF, and procaspase-3. SDS-PAGE loading controls were provided by the immunoblotting detection of β-actin. All error bars represent the SEM. *P < 0.01 between Scr vs. Scr + Q, PARG vs. PARG + Q, PARG + Q vs. PARG + Q + AIF, PARG + Q + AIF vs. PARG, and PARG + Q + AIF vs. Scr + Q (one-way ANOVA and unpaired Student’s t-test).

Since increased levels of PAR due to the RNAi silencing or genetic knockout of PARG led to decreased caspase-3/7/8/9 activity, we next investigated the effect of pan-caspase inhibition on cell death in PARG-silenced MDA-MB-231 cells after DNA-damaging treatments. Interestingly, the results showed that the pretreatment of PARG-silenced cells with Q-VD-OPh lead to an increase in MDA-MB-231 cell death after MNNG treatment (Figure 5A). This level of cell death was comparable to the amount of cell death observed in control cells treated with MNNG, as PARG-silenced cells pretreated with Q-VD-OPh (49%), untransfected cells (48%), and cells transfected with scrambled RNAi oligos (55%) all produced similar levels of cell death after MNNG treatment (Figure 5A). These results suggest that an alternative cell death pathway is induced after the silencing of PARG and the inhibition of caspases, such that it can substitute as the cell death executioner after MNNG treatment in the absence of caspase activity.

To identify this alternative cell death pathway in the absence of caspase activity, we performed the silencing of AIF by RNAi in PARG-silenced MDA-MB-231 cells pretreated with Q-VD-OPh. The results demonstrate that the silencing of AIF in these cells led to a profound amount of protection from MNNG treatment, since the amount of cell death observed was only 16% (Figure 5A). This was significantly decreased as compared to the level of cell death in PARG-silenced cells pretreated with Q-VD-OPh (50%). This indicates that AIF is a primary mediator of cell death that occurs after the inhibition of caspase activity in PARG-silenced cells. Further, the decrease in cell death due to AIF silencing (66%) was greater than the decrease in cell death observed in PARG-silenced cells (43%) or control cells (cells transfected with scrambled RNAi oligos) pretreated with Q-VD-OPh (49%). This indicates that AIF is responsible for mediating the majority of cell death that is observed after chemotherapy in PARG-silenced MDA-MB-231 cells in the absence of caspases.

A closer inspection of cell death in MDA-MB-231 cells after knockdown or inhibition of caspases, PARG, and AIF was performed by analyzing the amount of cells that exhibited propidium iodide (PI) and/or annexin V-FITC fluorescence after FACS analysis. The results revealed that the inhibition of caspases in control cells (via pretreatment with Q-VD-OPh) and the silencing of PARG by siRNA led to a decreased amount of cells that exhibited dual PI/annexin V fluorescence (Figure 5B). This indicates decreased levels of caspase-mediated apoptotic cell death. However, the combination of PARG-silencing and Q-VD-OPh treatment led to increased PI/annexin V staining, which would normally indicate increased levels of apoptosis. However, caspase inhibition via Q-VD-OPh pretreatment was confirmed by immunoblot, as procaspase-3 levels were decreased after MNNG treatment (due to caspase-3 activation), but were not decreased after the pretreatment of PARG-silenced cells with Q-VD-OPh (Figure 5C). This demonstrates that caspase-mediated apoptosis was not the primary type of cell death induced in PARG-silenced cells pretreated with Q-VD-OPh. The addition of AIF-silencing in these cells led to decreased levels of PI/annexin V staining (Figure 5B). The silencing of PARG and AIF protein levels by RNAi were confirmed by immunoblot (Figure 5C). Since AIF-mediated necroptosis also leads to the dual staining of cells with PI/annexin V [5], these results thus demonstrate that AIF is the primary mediator of cell death in PARG-silenced cells pretreated with Q-VD-OPh. Taken together, these results demonstrate that AIF can substitute as the primary mediator of cell death in PARG-silenced MDA-MB-231 cells in the absence of caspases, and it potentially identifies a critical role for AIF in the death of breast adenocarcinoma cells after chemotherapy.

## Discussion

Our results identify a potentially novel role for AIF in the death of breast cancer cells after chemotherapy. Here, we demonstrated a role for AIF in the death of breast adenocarcinoma cells after treatment with cisplatin, cyclophosphamide, or epirubicin, all currently utilized chemotherapeutic agents used to treat breast cancer [28,29]. The potential importance of AIF in breast cancer cell death is also seen in its ability to mediate cell death in the absence of caspase activity. Previously, it was shown that AIF can substitute as caspase executioner in ischemicneuronal injury mediated by N-methyl-D-aspartate (NMDA) receptor stimulation after the inhibition of caspase activity [18]. Likewise, the results here demonstrate the ability of AIF to substitute as caspase executioner in breast cancer cell death after increasing PAR levels and pretreatment with the pan-caspase inhibitor, Q-VD-OPh. The ability of AIF to mediate caspase dependent or caspase independent cell death is controversial. Initial studies demonstrated that the ability of AIF to induce cell death was caspase-independent [7,18], while subsequent studies demonstrated it was caspase-dependent [9,10]. This study provides further evidence for the ability of AIF to induce cell death in the absence of caspase activity. Thus, this paper presents evidence that AIF is an important mediator of breast cancer cell death after chemotherapy in the absence of caspases, and it also identifies a role for AIF in the mediation of caspase-independent cell death in breast adenocarcinoma cells.

These results reveal the potential ability for the pharmacologic activation of AIF to improve the chemotherapeutic treatment of breast cancer. Because of the dependence of nuclear AIF translocation on the synthesis of PAR, a feasible strategy to induce AIF-mediated cell death would appear to be elevating PAR levels by inhibiting PARG, the primary enzyme that catalyzes the hydrolysis of PAR, to improve the chemotherapeutic response. The modulation of PAR levels was shown to primarily affect rapidly dividing cells [33], which demonstrates the ability of the targeting of PAR to selectively target cancer cells. Our previous studies suggest that enhancing the synthesis of PAR may provide an improved strategy to successfully treat breast tumors due to the ability of PAR to activate AIF-mediated cell death. However, our data here shows that the silencing of PARG by RNAi leads to increased breast cancer cell survival. This is in agreement with a previous study that demonstrated that the silencing of PARG by RNAi in murine embryonic fibroblasts decreased cell death following hydrogen peroxide (H_2_O_2_) treatment [34]. However, most studies regarding the genetic disruption or knockdown of PARG demonstrate increased cell death. Stable knockdown of PARG by short hairpin RNA (shRNA) caused increased cell death following apoptotic doses of H_2_O_2_[35] and radiosensitivity to X-rays [36]. Genetic disruption of the *Parg* gene causes embryonic lethality and increased cell death in response to low doses of DNA-damaging agents [37,38]. Recently, we demonstrated the specific induction of AIF-mediated cell death in PARG-null cells after UV-C radiation [15]. Thus, the knockdown or genetic disruption of PARG is known to have a deleterious effect on cancer cells.

However, we provide evidence that PARG activity has a role in increasing caspase activity, such that the knockdown or absence of PARG leads to decreased levels of caspase activity and decreased levels of apoptotic cell death. This suggests that PARG may have multiple roles in the regulation of caspase-dependent and caspase-independent cell death. The difference in cell death versus cell survival after the silencing or absence of PARG may reflect differential roles for PARG isoforms on the modulation of cell death. The *Parg* gene is unique, but alternative splicing leads to five different PARG isoforms that localize to different cellular compartments [39]. Although the function of each is poorly understood, studies have identified a role for the nuclear isoform in DNA repair and the maintenance of genomic integrity [40], and a role for the cytoplasmic PARG isoforms to regulate the translation and stability of mRNAs after cell stress[41]. It is therefore possible that each PARG isoform may have a role in different pathways of cell death. Thus, while the nuclear PARG isoform may have a role in genomic stability and the regulation of caspase-independent cell death, the cytoplasmic isoforms may have roles in regulating caspase function in the cytosol in response to cell stress. Therefore, the specific inhibition of the nuclear PARG isoform may be required to optimize cell death in breast cancer cells in response to chemotherapeutic treatments.

This study utilized MNNG, which is an experimental chemotherapeutic agent only. However, it is a DNA-methylating agent that also induces free radical-mediated oxidative stress[42], which is similar to the mechanism of other DNA-alkylating chemotherapeutic agents[43]. It is also a well-established inducer of PAR metabolism and AIF-mediated cell death[12,33]. Although MNNG is not clinically utilized, it is an appropriate agent to utilize in order to study the molecular pathways of cell death in breast cancer cells mediated by PAR. Further, the similar mechanism of action that MNNG shares with other currently utilized DNA-alkylating chemotherapeutic agents indicates that MNNG is suitable for initial studies regarding the chemotherapeutic relevance of PAR/AIF in breast cancer cells. However, future preclinical studies expanding on our findings will be required to utilize currently used clinical agents.

Finally, the results provide novel insight into the ability of PAR to potentially regulate apoptosis. PAR was initially associated with caspases due to the specific cleavage of PARP-1 into 24 and 89 kDa forms by caspase 3 during apoptosis [44]. However, cells lacking PARP-1 were shown to undergo caspase-dependent apoptosis normally following stimuli that activate intrinsic or extrinsic apoptotic pathways [45]. Thus, a direct role for PAR in caspase-dependent cell death was not known. Here, we demonstrate the ability of PAR to negatively regulate caspase activity. Because PAR was shown to induce the translocation of AIF to the nucleus following genotoxic stress [12], it is possible that the absence of PARG, which leads to elevated levels of PAR, leads to the modulation of caspase activity and activates the nuclear translocation of AIF. Such a scenario, which requires further investigation, would provide novel insight into the mechanism of how cell death induced by PAR and mediated by AIF is caspase-independent.

## Conclusions

This study demonstrates a role for the pro-cell death mediator, AIF, in breast cancer cell death after chemotherapy. Further, the results demonstrate the novel ability of the hydrolysis of PAR by PARG to regulate caspase activity. We therefore report novel roles for AIF and PAR in the death of breast adenocarcinoma cells after chemotherapy. Moreover, we report the ability of AIF to substitute as primary mediator of breast cancer cell death in the absence of caspases after chemotherapy. Thus, these results identify the relevance of AIF in breast cancer cell death and they suggest that cell death by PAR/AIF may represent a strategy to optimize the eradication of breast tumors by activating an alternative cell death pathway.

## Methods

### Reagents

Dulbecco’s modified Eagle’s medium (DMEM) was purchased from Hyclone (Logan, UT). OptiMEM reduced serum medium was purchased from Gibco (Grand Island, NT). Fetal bovine serum (FBS) was purchased from Atlas Biologicals (Fort Collins, CO). Protease inhibitor cocktail tablets (Complete Mini, EDTA-free) were from Roche (Mannheim, Germany). Caspase inhibitor, Q-VD-OPh, was purchased from R&D Systems (Minneapolis, MN). Lipofectamine2000 reagent, trypsin, penicillin-streptomycin solution, and glutamine were purchased from Invitrogen (Carlsbad, CA). *N*-Methyl-*N*’-nitro-*N*-nitrosoguanidine (MNNG) was purchased from AccuStandard (New Haven, CT). Epirubicin, cisplatin and cyclophosphamide were all purchased from Calbiochem (La Jolla, CA). ApoScreen annexin V-fluorescein isothiocyanate (FITC) conjugate and propidium iodide (PI) were purchased from Southern Biotech (Birmingham, AL). Primary antibodies utilized were polyclonal anti-PAR clone 96–10 (Trevigen, Gaithersburg, MD), polyclonal anti-β-actin (Thermo Fisher Scientific), polyclonal anti-caspase-3 (Millipore, Billerica, MA), monoclonal anti-PARG (Millipore), polyclonal anti-AIF (Rockland Immunochemicals, Gilbertsville, PA), polyclonal anti-manganese superoxide dismutase (MnSOD) (Millipore), monoclonal anti-Lamin B2 clone LN43 (Thermo Fisher), and monoclonal anti-caspase-8 (Cell Signaling, Danvers, MA). Secondary antibodies horseradish peroxidase (HRP)-conjugated goat anti-rabbit and anti-mouse were purchased from Sigma (St. Louis, MO).

### Cell Culture

Human breast adenocarcinoma cells (MDA-MB-231 and MCF-7) were cultured in DMEM supplemented with 10% FBS, 2 mM L-glutamine and 100U/ml penicillin/streptomycin at 37°C in 5% CO_2_. Wild type and PARG-null primary embryonic trophoblast stem (TS) cells were derived from E3.5 mouse blastocysts and cultured as previously described [33]. For siRNA transfections, each cell line was cultured at least one day in antibiotic-free medium before transfection.

### RNA interference

The silencing of AIF by RNAi was performed as previously reported using sense (5’-CUUGUUCCAGCGAUGGCAUtt-3’) and antisense (5’-AUGCCAUCGCUGGAACAAGtt-3’) RNA oligos that target nucleotides 151–171 in human AIF [26]. The silencing of PARG by RNAi was performed as previously reported using RNA oligos 5’-AAATGGGACTTTACAGCTTTG-3’) that target nucleotides 1960–1980 in human PARG [34]. Control oligos 5’-AGACAGAAGACAGAUAGGCtt-3’ (sense) and 5’-GCCUAUCUGUCUUCUGUCUtt-3’ (antisense) were used for all negative controls [26]. All RNA oligos were purchased as duplexed RNA from Sigma-Aldrich (St. Louis, MO). Each siRNA duplex was resuspended in RNAase-free H_2_O at a final concentration of 20 μM and stored at −20°C.

For RNAi transfections, cells were plated in 0.5 ml medium per well without antibiotics in 24-well plates one day before transfection. At the time of transfection, cells were approximately 50% confluent. For each transfection, duplex siRNA was added to 50 μl OptiMEM medium and 1 μl lipofectamine2000 was added to 50 μl OptiMEM. After 5 min, the two solutions were gently mixed and then incubated at room temperature for 20 min. Final concentrations of siRNA were 20 nM for AIF and 40 nM for PARG. The mixtures were added to each well and cells were cultured for an additional 48–72 h.

### Whole cell lysate extraction

Cells were harvested by trypsinization, washed with PBS, suspended in lysis buffer containing 10 mM Tris–HCl (pH8), 0.1 M NaCl, 1 mM EGTA, 1 mM EDTA, 0.1% triton X-100, and protease inhibitors (Complete Protease Inhibitor Cocktail tablets, Roche). Extracts were then incubated on ice for 30 min with vortexing every 10 min. Cleared cell lysates were obtained after centrifugation at 16,000 xg for 10 min. SDS-PAGE sample buffer was added to supernatants to achieve final concentrations of 50 mM Tris–HCl with pH 6.8, 1% SDS, 2.5% glycerol, 0.002% bromophenol blue, and 143 mM 2-mercaptoethanol. Samples were then boiled for 2 min at 100°C.

### Subcellular Fractionations

Cells were harvested by trypsinization and washed with PBS. Pellets were fractionated using the NE-PER Nuclear and Cytoplasmic Extraction Kit (Pierce, Rockfield, IL) according to the manufacturer’s protocol. Nuclear and cytoplasmic fractions were prepared in SDS sample buffer and boiled as previously described.

### Immunoblotting

Approximately 20 μg of each lysate or subcellular sample was subjected to 8-12% sodium dodecyl sulfate polyacrylamide gel electrophoresis (SDS-PAGE). The proteins were transferred to nitrocellulose by semi-dry transfer at 25 V for 1 h using a Trans-blot SD apparatus (BioRad, Hercules, CA). Membranes were blocked with PBS containing 5% milk and 0.1% Tween-20 (PBST) at room temperature for 1 h and incubated with primary antibodies (1:2000 anti-AIF, 1:2000 anti-PARG, 1:3000 anti-MnSOD, 1:1000 anti-LaminB2, 1:10,000 anti-PAR, 1:10000 anti-beta-actin, 1:2000 anti-caspase3 or 1:2000 anti-caspase-8) in PBST + 5% milk overnight (shaking) at 4°C. Membranes were then washed with PBST three times and incubated with horseradish peroxidase (HRP)-conjugated goat anti-rabbit or anti-mouse antibody (1:10000) for 1 h. The membranes were washed as described above, and chemiluminescence was initiated using the Supersignal West Pico reagents (Pierce). Immunoblots were then developed on a ChemiDoc XRS gel imaging system (Bio-Rad). For quantification of protein levels for each blot, immunoblots were examined by densitometry with the ChemiDoc imager using Quantity One software. Relative densitometry ratios were then calculated using β-actin as loading controls. The resulting AIF/actin, PARG/actin, or procaspase-8/actin values were then used to quantify relative protein levels.

### Cell Viability Assay

Forty-eight hours after cells were transfected with siRNA, cells were treated with 0.5 mM MNNG, an experimental chemotherapeutic agent that methylates DNA [42] and is known to induce PAR-mediated cell death [12,33,46]. Cells were also treated with 20 μM epirubicin (EPI), a dose similar to those used in MDA-MB-231 breast cancer cells in previous reports [47,48] and consistent with the reported IC_50_ of this agent in breast cancer cells (55.3 μM) [49]. Other treatments consisted of 10 mM cyclophosphamide (CPA), a dose previously utilized in MDA-MB-231 breast cancer cells [50], or 0.5 mM cisplatin (DDP), an elevated dose for this agent, but the exposure time utilized (6 hr) was 4 to 12-fold less than normally indicated (24–72 hr). Further, this dose of DDP was consistent with the reported IC_50_ of this agent in breast cancer cells (0.6 mM) [51]. Twenty hours after treatment, cells were harvested, washed with PBS, and resuspended in annexin-binding buffer (10 mM HEPES, pH 7.4, 140 mM NaCl, 2.5 mM CaCl_2_,) to approximately 1 X 10^6^ cells/ml. For each 100 μl of cell suspension, 2.5 μl of ApoScreen annexin V-FITC conjugate solution (Southern Biotech) and 1 μl of 100 μg/ml propidium iodide (PI) solution was added. Cells were then incubated at room temperature for 15 min and 400 μl annexin-binding buffer was added. Cell death was then measured by quantifying the percentage of cells that exhibited annexin V-FITC and/or PI fluorescence using a flow cytometer (Accuri, Ann Arbor, MI). Total cell death was quantified by adding the percentage of cells detected in the upper left (PI), upper right (PI + annexin V-FITC), and lower right (annexin V-FITC) quadrants in the FACS dot plots. For pretreatment with Q-VD-OPh, cells were treated with 40 μM Q-VD-OPh 30 min prior to chemotherapeutic treatments. Treatment with Q-VD-OPh continued during all treatments, and until FACS analyses were performed.

### Caspase Activity Assay

Wild type and PARG null TS cells were cultured and exposed to 25 J/m^2^ UV-C using a low-pressure Hg lamp (model G30T8, Sylvania, Danvers, MA). 12 h after UV exposure, 1 X 10^5^ cells were seeded in 96-well opaque black plate and subjected to Apo-ONE Homogeneous Caspase-3/7, Caspase-8, and Caspase-9 Assays (Promega) according the manufacturer’s specifications. Fluorescence was analyzed using a Bio-Tek (Winooski, VT) Synergy HT plate reader at an excitation wavelength of 485 nm and at an emission wavelength of 527 nm. Negative controls were provided by untreated cells and cells pretreated with 40 μM Q-VD-OPh.

### Statistical analyses

All error bars for calculated cell death, caspase activity, and protein levels represented the standard error of the mean (SEM). Statistical analyses were accomplished by one-way analysis of variance (ANOVA) and unpaired Student’s t-test.

## Abbreviations

AIF: apoptosis-inducing factor; CPA: cyclophosphamide; DDP: cisplatin; EPI: epirubicin; FITC: fluorescein isothiocyanate; H_2_O_2_: hydrogen peroxide; MNNG: N-methyl-N’-nitro-N-nitrosoguanidine; MnSOD: manganese superoxide dismutase; NADH: nicotinamide adenine dinucleotide (reduced form); NMDA: N-methyl-D-aspartate; PAR: poly(ADP-ribose); PARG: poly(ADP-ribose) glycohydrolase; PARP: poly(ADP-ribose) polymerase; PI: propidium iodide; Q-VD-OPh: N-(2-Quinolyl)valyl-aspartyl-(2,6-difluorophenoxy)methyl ketone; RNAi: RNA interference; shRNA: short-hairpin RNA; siRNA: small-interfering RNA.

## Competing interests

No conflict of interest by any of the authors is present due to this work.

## Authors’ contributions

XF carried out the RNAi studies, cell death studies, flow cytometry studies, participated in the design of the study, and helped draft the manuscript. YZ carried out the caspase activity experiments and participated in the design of the study. AMP carried out the flow cytometry experiments. MMH and ML carried out the flow cytometry studies and participated in the design of the study. DWK conceived of the study, participated in its design and coordination, and helped to draft the manuscript. All authors read and approved the final manuscript.
